# *Piper cubeba* L. Methanol Extract Has Anti-Inflammatory Activity Targeting Src/Syk via NF-κB Inhibition

**DOI:** 10.1155/2019/1548125

**Published:** 2019-01-01

**Authors:** Nurinanda Prisky Qomaladewi, Nur Aziz, Mi-Yeon Kim, Jae Youl Cho

**Affiliations:** ^1^Department of Integrative Biotechnology, Sungkyunkwan University, Suwon 16419, Republic of Korea; ^2^School of Systems Biomedical Science, Soongsil University, Seoul 06978, Republic of Korea

## Abstract

*Piper cubeba *L. is a plant in the Piperaceae family that is generally found in tropical countries and acts as an antioxidant and anti-inflammatory agent. Unfortunately, the molecular mechanism of the anti-inflammatory activity has not been fully investigated. In this study, we elucidated the anti-inflammatory mechanism by focusing on NF-*κ*B signaling, which is considered a prototypical inflammatory signaling pathway in both innate and adaptive immune functions. Cellular activity and the molecular target of Pc-ME were identified in macrophage RAW264.7 cells and HEK293T cells by assessing NO production, cytokine expression by RT-PCR, luciferase gene reporter assay, and protein regulation in cytoplasm by Western blot upon NF-*κ*B activation. Pc-ME reduced NO production without any cell toxicity; inhibited expression of proinflammatory cytokines such as iNOS and IL-6; downregulated NF-*κ*B activation mediated by both MyD88 and TRIF; and diminished the phosphorylation of I*κ*B*α*, IKK*α*/*β*, Akt, p85, Src, and Syk. Pc-ME inhibited Syk and Src autophosphorylation during overexpression in HEK cells, which confirmed our hypothesis that Syk and Src were signaling targets of Pc-ME. These findings indicate that* Piper cubeba *L. has anti-inflammatory activity by targeting Src/Syk in the NF-*κ*B pathway.

## 1. Introduction

Inflammation is a protective strategy in response to insults such as microbial infection, tissue injury, and other noxious conditions. It causes several classical symptoms, including redness, pain, swelling, and heat [1]. In the innate immune system, host cells express various pattern recognition receptors (PRRs) that sense diverse pathogen-associated molecular patterns (PAMPs). Toll-like receptors (TLRs), which act as PRRs in mammals, activate signaling pathways that induce expression of several genes involved in the immune response [2, 3]. Upon recognizing PAMPs, TLRs dimerize and activate signaling pathways that originate from conserved cytoplasmic TIR (Toll-Interleukin 1 Receptor) domain-containing adaptors, including MyD88 and TRIF [4]. These adaptors connect external stimuli to intracellular signaling molecules such as protein tyrosine kinases [Syk and Src], serine/threonine kinases [AKT, phosphatidylinositide 3 kinases (PI3K), and I*κ*B kinase (IKK)] and activate transcription of inflammatory genes such as iNOS, interleukin- (IL-) 6, and other cytokines by activating transcription factors such as nuclear factor-*κ*B (NF-*κ*B) [5].

*Piper cubeba *L. is a Piperaceae plant that is indigenous to tropical countries, especially South Borneo and Indonesia. Important constituents of cubebs are volatile oil, cubebic acid, cubeb resin, and lignans. Some of its lignans, e.g., cubebin and hinokinin, have been reported to have anti-inflammatory and analgesic effects [6].* In vivo*, a crude hydroalcoholic extract had anti-inflammatory and analgesic properties [7]. Some reports also found that* Piper cubeba *L. extract has anti-inflammatory activity by attenuating IL-6, which is stimulated by lipopolysaccharide (LPS), in THP-1 cells resulting in inhibition of cyclooxygenases (COX-1 and COX-2) and 5-lipoxygenase (5-LOX) [7]. A study also found that a crude extract of* Piper cubeba *L. has antioxidant activity, which could treat skin inflammation caused by radicals such as ROS and hydrogen peroxide [8].

Although previous studies have shown that* Piper cubeba *L. has anti-inflammatory activity, the exact molecular target of this activity has not been investigated. Here we investigated the anti-inflammatory activity by using LPS to stimulate TLR4* in vitro*. We identified the molecular targets of this extract with various molecular and biochemical approaches.

## 2. Materials and Methods

### 2.1. Materials

Methanol extract of* Piper cubeba *(Pc-ME) was purchased from the Plant Extract Bank at the Plant Diversity Research Center (Daejeon, Korea; http://extract.kribb.re.kr/extract/f.htm, e-mail: mplantext@kribb.re.kr). The cell lines used in these experiments were from ATCC (Rockville, MD, USA). RAW264.7 cells are a BALB/c-derived murine macrophage cell line, and HEK293T cells are a human embryonic kidney cell line. Cell culture products, such as fetal bovine serum (FBS), penicillin/streptomycin, DMEM, and RPMI1640, as well as TRIzol were purchased from Gibco Products (Grand Island, NY, USA). Phosphate-buffered saline (PBS) was from Capricorn Scientific (Ebsdorfergrund, Germany). Polyethylenimine (PEI), Pam3CSK4, lipopolysaccharide (LPS, Escherichia coli 0111:B4), tetrazole 3-(4,5-dimethylthiazol-2-yl)-2,5-diphenyltetrazolium bromide (MTT), sodium dodecyl sulfate (SDS), and dimethyl sulfoxide (DMSO) were obtained from Sigma Chemical Co. (St. Louis, MO, USA). RT-PCR primers were from Bioneer (Seoul, South Korea). Luciferase constructs with NF-*κ*B binding promoter sites were used as previously reported [5]. Phospho-specific and total antibodies against p50, p65, I*κ*B*α*, I*κ*B kinase (IKK) *α*/*β*, Akt (Ser 473), p85, Syk, Src, and *β*-actin were purchased from Cell Signaling Technology (Beverly, MA, USA) and Santa Cruz Biotechnology (Santa Cruz, CA, USA). PP2 and piceatannol (Picea) were from Calbiochem (La Jolla, CA, USA).

### 2.2. Cell Culture and Drug Preparation

RAW264.7 and HEK293T were cultured in RPMI1640 and DMEM media, respectively, supplemented with 1% penicillin/streptomycin, and 10% (RPMI1640) or 5% (DMEM) FBS. The cells were grown at 37°C and 5% CO_2_. Stock solution of Pc-ME was prepared by diluting in concentration of 100 mg/ml using DMSO.

### 2.3. Determination of NO Production

RAW264.7 cells (1x10^6^ cells/ml) were preincubated for 18 h [9], treated with Pc-ME (0-100 *μ*g/ml) or prednisolone as a positive control (0-200 *μ*M) for 30 min, and then treated with LPS (1 *μ*g/ml) or Pam3CSK4 (10 *μ*g/ml), respectively, for 24 h. The inhibitory effects of Pc-ME and prednisolone on NO production were determined with Griess reagent, as previously described [10].

### 2.4. Cell Viability Assay

RAW264.7 cells (1x10^6^ cells/ml) were preincubated for 24 h then treated with Pc-ME (0-100 *μ*g/ml) or prednisolone (0-200 *μ*M). The cytotoxic effects of Pc-ME and prednisolone were evaluated with an MTT assay as previously reported [11].

### 2.5. HPLC Analysis

High-performance liquid chromatography (HPLC) analysis was used to confirm the phytochemical characteristics of Pc-ME with standard compounds including, quercetin, luteolin, and kaempferol [12], as previously reported [13, 14].

### 2.6. Analysis of mRNA Levels by Reverse Transcription-Polymerase Chain Reaction

To determine levels of cytokine mRNA expression, RAW264.7 cells were treated with Pc-ME (50 and 100 *μ*g/ml) for 30 min and then with LPS (1 *μ*g/ml) for an additional 6 h. Total RNA was extracted with TRIzol Reagent (Gibco RBL) according to the manufacturer's instructions, and the isolates were kept at -70°C until use. Semiquantitative RT-PCR was conducted as previously reported [15]. The primers (Bioneer, Seoul, Korea) used are listed in Table 1.

### 2.7. Preparation of Cell Lysates and Nuclear Fractions for Immunoblotting

After being treated with Pc-ME for 30 min, RAW264.7 cells (2x10^6^ cells/ml) were treated with LPS for the indicated times. The cells were washed with cold PBS and lysed in lysis buffer (20 mM Tris-HCl, pH 7.4; 2 mM ethyleneglycotetraacetic acid; 50 mM *β*-glycerophosphate; 1 mM sodium orthovanadate; 1 mM dithiothreitol; 1% Triton X-100; 10% glycerol; 10 *μ*g/ml aprotinin; 10 *μ*g/ml pepstatin; 1 mM benzamide; and 2 mM PMSF) and centrifugation at 12,000 rpm for 15 min at 4°C. The whole cell lysates were stored at -70°C until use. To collect nuclear lysates, RAW264.7 cells (5x10^6^ cells/ml) were treated with Pc-ME and LPS and washed in cold PBS then washing buffer, which contains 10 mM HEPES (pH 7.8) and KCl, 2 mM MgCl_2_. 0.1 mM EDTA and PMSF, 1 mM DTT, and 2 *μ*g/ml leupeptin and aprotinin. The cells were lysed in washing buffer with 10% Nonidet P-40 then centrifuged at 14000 rpm. The pellet (nuclear fraction) was washed in the same buffer, and nuclei were treated with extraction buffer consisting of washing buffer with 50 mM KCl, 0.3 M NaCl, 10% glycerol. The extract was centrifuged at 14,000 rpm for 5 min and the supernatant was collected as the nuclear fraction and stored at -70°C until use. Soluble cell lysates were analyzed by immunoblotting. Proteins were separated on 10% SDS-polyacrylamide gels and transferred by electroblotting onto a polyvinylidenedifluoride (PVDF) membrane. After the transfer, the membranes were blocked for 60 min in Tris-buffered saline containing 3% BSA and 0.2% Tween 20 at room temperature. The membranes were incubated at room temperature for 60 min with primary antibodies, washed three times (10 min each) with the same buffer, and incubated for 60 min with HRP-conjugated secondary antibodies. The total and phosphorylated levels of p65 (MW: 65), p50 (50), I*κ*B*α* (40), IKK*α*/*β* (85/87), AKT (60), p85 (60-85), Syk (72), Src (60), and *β*-actin (45) were visualized with an ECL system (Amersham, Little Chalfont, Buckinghamshire, UK) as previously reported [16].

### 2.8. Plasmid Transfection and Luciferase Reporter Gene Assay

HEK293T cells (2x10^5^ cells/ml) were cultured for 18 h before transfection with plasmids (0.8 *μ*g/ml each well) encoding a luciferase gene under an NF-*κ*B promoter. Cells were cotransfected with MyD88 and TRIF by the polyethylenimine (PEI) method. After a 24-h stabilization, the transfected cells were treated with Pc-ME for 24 h. Luciferase activity was assessed with the Luciferase Assay System (Promega, Madison, WI, USA) as previously reported [17]. To evaluate Src and Syk autophosphorylation, HEK293T cells (1x10^6^ cells/ml) were transfected with genes encoding Src and Syk gene for 24 h. The cells were treated with Pc-ME for 24 h. The levels of phosphorylated Src and Syk, HA and Myc (tag protein), and *β*-actin were visualized from whole cell lysates of Src- and Syk-transfected cells by immunoblot analysis.

### 2.9. Statistical Analysis

All data in this study are presented as mean ± standard deviation (SD) calculated from three samples. For statistical comparison, all values were analyzed with ANOVA/Scheffe's post hoc test as well as the Kruskal-Wallis/Mann-Whitney test. A* P-*value < 0.05 or < 0.01 was accepted as statistically significant. Statistical evaluation was determined with SPSS software (SPSS Inc., Chicago, IL, USA). Similar experimental data were obtained from an additional independent set of in vitro experiments performed under the same conditions.

## 3. Results

### 3.1. Effect of Pc-ME on NO Production

**Figure 1:**
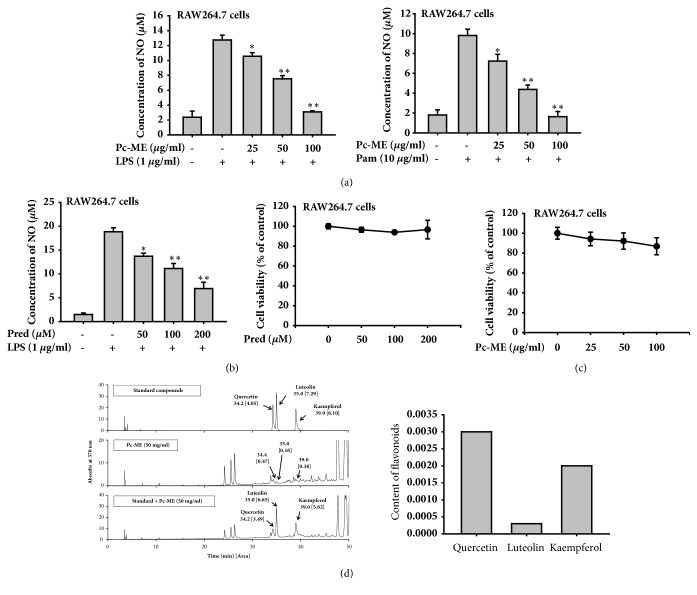
**Effect of Pc-ME on NO production in LPS-induced macrophages. **(a) NO production assay in RAW264.7 cells (10^6^ cells/ml) induced with 1 *μ*g/ml of LPS (left panel) or 10 *μ*g/ml of Pam3CSK4 (Pam, right panel) for 24 h. (b) NO production after prednisolone (Pred) treatment as a positive control (0-200 *μ*M) (left panel) and viability of RAW264.7 cells after prednisolone treatment (right panel). (c) Viability of RAW264.7 cells after Pc-ME treatment (0-100 *μ*g/ml) assessed with MTT solution. (d) Phytochemical profile (left panel) and the content (right panel) of flavonoids (quercetin, luteolin, and kaempferol) were obtained by HPLC analysis. *∗p *< 0.05 and *∗∗p *< 0.01 compared to control or normal groups. All data (a–c) are expressed as mean ± SD of 3 replicates.

To evaluate the effects of Pc-ME on NO (nitric oxide) as an inflammatory mediator, we stimulated murine macrophages (RAW264.7 cells) with LPS and Pam3CSK4 as inflammatory inducers, and treated cells with Pc-ME at a range of concentrations (0 to 100 *μ*g/ml). With LPS as an inducer, Pc-ME extract reduced NO production by more than 80% at 100 *μ*g/ml (Figure 1(a) left panel). NO production can also be induced by TLR1/2 recognizing Pam3CSK4. Pc-ME (100 *μ*g/ml) inhibited NO production induced by 10 *μ*g/ml Pam3CSK4 by more than 80% (Figure 1(a) right panel). For comparison, we used prednisolone, a common anti-inflammatory drug, which only inhibited approximately 40% of NO production at 200 *μ*M (Figure 1(b) left panel). Cell viability assays were performed to verify that Pc-ME was not toxic to cells at the concentrations used. Cell viability remained at more than 80% of untreated cells for Pc-ME concentrations up to 100 *μ*g/ml after 24 h (Figure 1(c)).

### 3.2. Investigation of Pc-ME Contents by HPLC

Investigation of Pc-ME by HPLC has shown that Pc-ME includes quercetin, luteolin, and kaempferol which are known as anti-inflammatory flavonoid compounds (Figure 1(d)).

### 3.3. Effects of Pc-ME on Expression of Proinflammatory Cytokines

**Figure 2:**
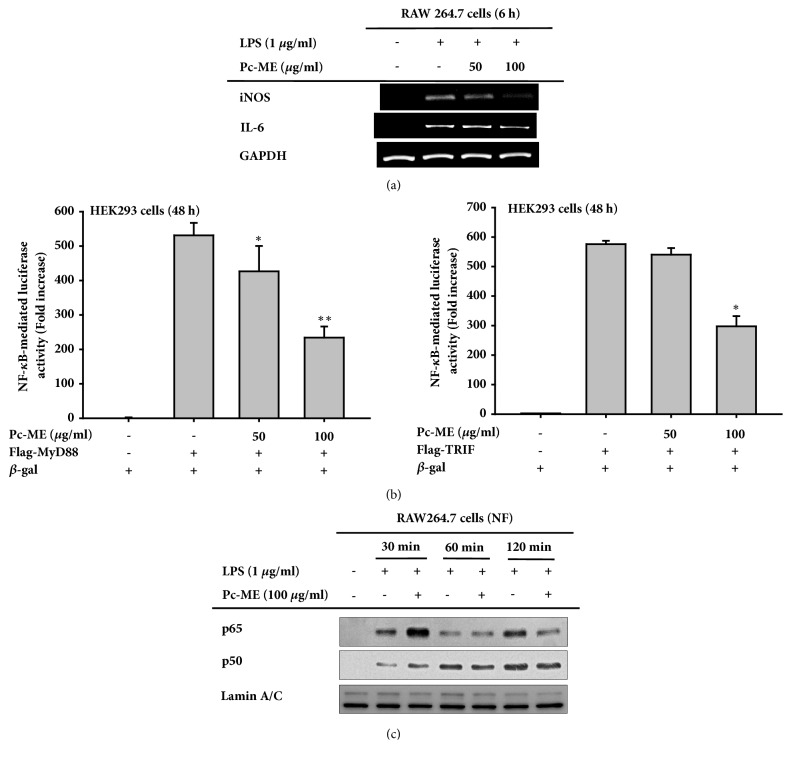
**Effect of Pc-ME on proinflammatory cytokines and NF-**κ**B transcription factor.** (a) Semiquantitative PCR for iNOS and IL-6 expression in RAW264.7 cells (2 x 10^6^ cells/ml)induced by LPS for 6 h with 50 and 100 *μ*g/ml of Pc-ME. (b) HEK 293 cells (2 x 10^5^ cells/ml) were cotransfected with NF-*κ*B-Luc, *β*-gal (0.8 *μ*g), and MyD88 or TRIF for 48 h with or without 50 and 100 *μ*g/ml of Pc-ME. (c) Immunoblot for p65 and p50 in the nuclear fraction of LPS-treated RAW264.7 cells (5 x 10^6^ cells/ml) with or without 100 *μ*g/ml of Pc-ME. *∗p *< 0.05 and *∗∗p* < 0.01 compared to control groups. All data (b) are expressed as mean ± SD 3 replicates.

Pc-ME of inflammation was also assessed by evaluating proinflammatory cytokines. Cells were treated with LPS for 6 h to upregulate mRNA levels, and 100 *μ*g/ml Pc-ME decreased expression of iNOS and IL-6 mRNA (Figure 2(a)). This result indicates that Pc-ME can inhibit transcription and translation of some inflammatory cytokines.

### 3.4. Effects of Pc-ME on Transcription Factor Activation

To investigate which proteins and transcription factors are targeted by Pc-ME as an anti-inflammatory agent, a luciferase reporter assay was carried out. As shown in Figure 2(b), 100 *μ*g/ml Pc-ME decreased NF-*κ*B-mediated expression of both MyD88 and TRIF by approximately 300 fold. AP-1-mediated expression of both proteins was not affected (data not shown).

### 3.5. Effects of Pc-ME on Activation of the NF-*κ*B Signaling Pathway

**Figure 3:**
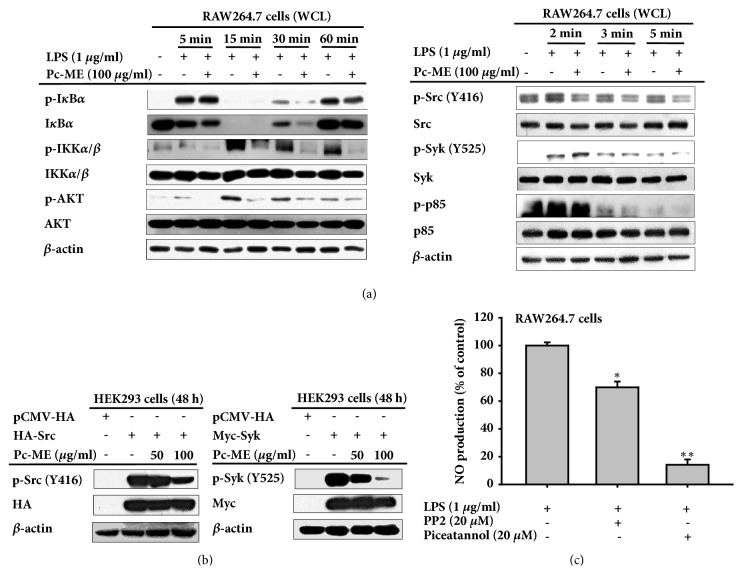
**Pc-ME anti-inflammatory effects on NF-**κ**B signaling.** (a) RAW264.7 cells (2 x 10^6^ cells/ml) were treated with LPS for various times with or without 100 *μ*g/ml of Pc-ME. Phosphorylated and total I*κ*B*α*, IKK*α*/*β*, AKT, p85, Syk, and Src were evaluated by immunoblot. (b) Syk and Src were transfected into HEK293 cells (2 x 10^5^ cells/ml) for overexpression with 50 and 100 *μ*g/ml of Pc-ME. Syk and Src phosphorylation were evaluated. (c) NO production with Syk and Src inhibitors, 20 *μ*M of piceatannol and PP2, respectively, in LPS-induced RAW264.7 cells (10^6^ cells/ml) within 24 h. *∗p *< 0.05 compared to control or normal groups. All data (c) are expressed as mean ± SD of 3 replicates. WCL: whole cell lysates.

Because Pc-ME was involved in NF-*κ*B activation, we evaluated the protein targets of NF-*κ*B activation by evaluating two NF-*κ*B subunits, p65 and p50/p105, in the nucleus. Nuclear fractions were collected to determine whether Pc-ME suppressed NF-*κ*B activity. After LPS induction for 120 min (Figure 2(c)), 100 *μ*g/ml Pc-ME suppressed expression of both NF-*κ*B subunits. We then evaluated the expression of upstream proteins involved in NF-*κ*B activation. Pc-ME also decreased the proportion of phosphorylated to total protein for I*κ*B*α*, IKK*α*/*β*, and AKT after 60 min LPS treatment (Figure 3(a)). Some proteins were assessed after shorter induction times to determine how Pc-ME affects proteins upstream of NF-*κ*B. After a 5-min treatment with LPS, Pc-ME suppressed p85, Syk, and Src activation in time-dependent manner. These findings were reinforced by overexpressing Syk and Src to ensure that they were targets of Pc-ME. As shown in Figure 3, 100 *μ*g/ml Pc-ME dramatically diminished both Syk and Src expression. To confirm whether Syk and Src were responsible for NO production through NF-*κ*B signaling, NO production was assessed after treatment with Syk and Src inhibitors, piceatannol and PP2, respectively. Compared to LPS, 20 *μ*M PP2 and piceatannol suppressed NO production, indicating that they have a role in inflammation by producing NO through NF-*κ*B signaling.

## 4. Discussion

This study was designed to investigate the anti-inflammatory effects of* Piper cubeba *L. methanol extract (Pc-ME). Innate and adaptive immunity strongly depend on TLRs recognizing pathogens through PAMPs, which leads to inflammation [18, 19]. Each TLR recognizes distinct PAMPs, for example TLR1, TLR2, and TLR6 recognize lipoproteins from gram-positive bacteria, and TLR4 recognizes lipopolysaccharide (LPS) from gram-negative bacteria [20, 21]. Here, we first screened the anti-inflammatory effects of Pc-ME by assessing NO after LPS and Pam3CSK stimulation of TLR4 and TLR 1/2, respectively [22, 23]. As shown in Figure 1(a), Pc-ME inhibited NO production induced by both gram-positive and -negative bacteria [24]. The ability of Pc-ME to reduce NO without affecting the cell was confirmed by measuring cell viability (Figure 1(c)). Anti-inflammatory activity is generally driven by the presence of polyphenols, especially flavonoids such as kaempferol, quercetin, apigenin, and luteolin. They are proven to inhibit proinflammatory cytokines expression [25]. By referring to these flavonoids, HPLC of Pc-ME presents to have some flavonoids' components such as quercetin, kaempferol, and luteolin, confirmed that Pc-ME potentially has an anti-inflammatory activity (Figure 1(d)).

Inflammatory responses are generally characterized by the activation of various signaling pathways. The NF-*κ*B signaling pathway in particular regulates expression of proinflammatory cytokines such as interleukin 1 (IL-1) and tumor necrosis factor (TNF)-*α* [26]. To demonstrate that Pc-ME is anti-inflammatory, expression of proinflammatory cytokine mRNA was measured by PCR. As shown in Figure 2(a), Pc-ME reduces iNOS and IL-6 mRNA levels, indicating that Pc-ME could inhibit transcription and translation of certain cytokines. Since Pc-ME could inhibit NO production and transcription of some proinflammatory cytokines induced by LPS and Pam3CSK4, a reporter gene assay and nuclear fractionation were conducted. The purpose of these assays was to determine which proteins and transcription factors are targeted by Pc-ME as an anti-inflammatory agent. Figure 2(b) shows that 100 *μ*g/ml Pc-ME decreased NF-*κ*B expression mediated by both MyD88 and TRIF, TLR adapter proteins, by approximately 300 folds. AP-1 expression mediated by both proteins did not change (data not shown).

NF-*κ*B plays a critical role in mediating inflammatory responses. It consists of two subunits, p65 and p50, that associate to form homo- and heterodimers with several roles including association with I*κ*B, nuclear translocation, and DNA binding [27]. Therefore, Pc-ME reduced both p65 and p50 levels in the nucleus. p65 is more crucial for NF-*κ*B activation in the inflammatory response, Figure 2(c) shows that p65 had a greater decrease at 120 min. Pc-ME could inhibit NF-*κ*B by reducing I*κ*B*α* phosphorylation, which began at 30 min (Figure 3(a)). We continued to assess upstream NF-*κ*B signaling molecules to determine the exact target of Pc-ME to inhibit inflammation. I*κ*B degradation activates the IKK complex, is required for NF-*κ*B activation, and is mediated by Akt [28, 29]. Pc-ME diminished LPS-induced expressions in a time-dependent manner (Figure 3(a) Left panel). In addition, we investigated signals upstream of Akt after short LPS exposure to determine the possible target of Pc-ME to inhibit inflammatory responses. Akt is phosphorylated by PI3K which is an upstream kinase for phosphorylating I*κ*B*α* [30]. The sarcoma (Src) tyrosine family kinase and spleen tyrosine kinase (Syk) are the main effector molecules involved in PRR-PAMP recognition [31, 32]. Early Src activation can mediate I*κ*B*α* phosphorylation resulting in NF-*κ*B activation [31]. Syk also has a role in phosphorylating p85 and Akt [33]. These associations indicate that Syk and Src are crucial for NF-*κ*B activation in inflammation. Therefore, we investigated the effects of Pc-ME extract in macrophage RAW264.7 cells during short exposure to LPS. As shown in Figure 3(a) right panel, Pc-ME diminished phosphorylation of the PI3K subunit p85 following Src and Syk activation at 3 and 5 min. Thus, we can conclude that Pc-ME might have anti-inflammatory activity by inhibiting Src or Syk kinase. To verify this finding, we overexpressed Src and Syk in HEK 293 cells, and 100 *μ*g/ml Pc-ME still diminish both Src and Syk phosphorylation. These findings were supported by using Src and Syk inhibitor, PP2 and piceatannol, respectively, to confirm that they regulate NO production in macrophage RAW264.7 cells.

**Figure 4:**
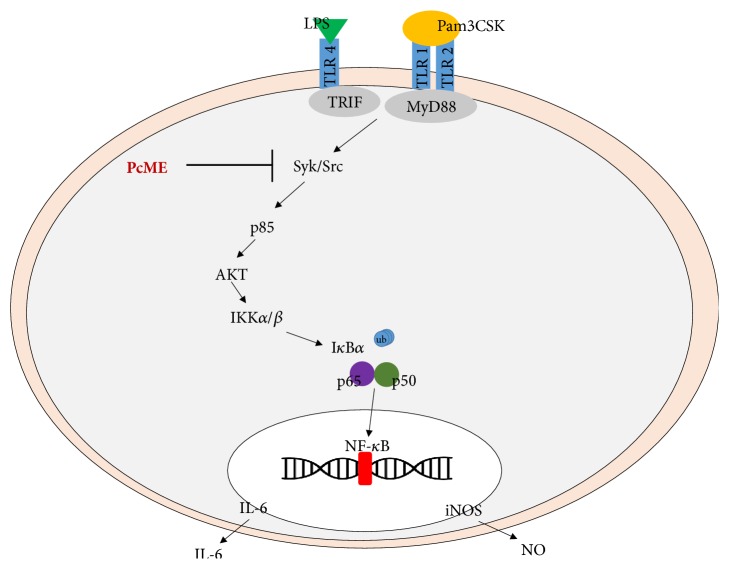
Possible Pc-ME targets in inhibiting NF-*κ*B signaling.

Conclusively, after investigating the molecular mechanism of Pc-ME in LPS-treated macrophage cells, we could suggest that Pc-ME is specifically able to inhibit inflammatory responses through NF-*κ*B by targeting Src/Syk (Figure 4). Future development of anti-inflammatory treatments from natural plants, peculiarly* Piper cubeba *L. extract, could be promising.

## Figures and Tables

**Table 1:** Sequences of PCR primers used in this study.

Name		Sequence (5' to 3')
Semi-quantitative PCR		

iNOS	F	GTGAAGAAAACCCCTTGTGCTG
	R	AGTTCCGAGCGCGTCAAAGACC

IL-6	F	GTACTCCAGAAGACCAGAGG
	R	TGCTGGTGACAACCACGG

GAPDH	F	ACCACAGTCCATGCCATCAC
	R	CCACCACCCTGTTGCTGTAG

## Data Availability

The data used to support the findings of this study are available from the corresponding author upon request.
